# Balloon Flower Root-Derived Extracellular Vesicles: In Vitro Assessment of Anti-Inflammatory, Proliferative, and Antioxidant Effects for Chronic Wound Healing

**DOI:** 10.3390/antiox12061146

**Published:** 2023-05-24

**Authors:** Manho Kim, Hyejun Jang, Ju Hyun Park

**Affiliations:** Department of Biomedical Science, Kangwon National University, Chuncheon-si 24341, Republic of Korea; manhokim@kangwon.ac.kr (M.K.); hyejunjang@kangwon.ac.kr (H.J.)

**Keywords:** antioxidants, balloon flower root, chronic wound healing, extracellular vesicles, oxidative stress

## Abstract

Excessive reactive oxygen species (ROS) in wound lesions can lead to oxidative stress and failure of normal wound healing processes, eventually resulting in chronic skin wounds. A multitude of researchers have investigated various natural products with physiological activities, including antioxidant effects, for healing chronic skin wounds. Balloon flower root (BFR), which contains bioactive components such as platycodins, is known for its anti-inflammatory and antioxidant effects. In this study, we isolated BFR-derived extracellular vesicles (BFR-EVs) that possess anti-inflammatory, proliferative, and antioxidant activities via a combination of polyethylene glycol-based precipitation and ultracentrifugation. Our objective was to investigate the potential of BFR-EVs in treating chronic wounds caused by ROS. Despite efficient intracellular delivery, BFR-EVs showed no significant cytotoxicity. In addition, BFR-EVs inhibited the expression of pro-inflammatory cytokine genes in lipopolysaccharide-stimulated RAW 264.7 cells. Furthermore, water-soluble tetrazolium salt-8 assay showed that BFR-EVs had a proliferation-promoting effect on human dermal fibroblasts (HDFs). Scratch closure and transwell migration assays indicated that BFR-EVs could promote the migration of HDFs. When the antioxidant effect of BFR-EVs was evaluated through 2′,7′-dichlorodihydrofluorescein diacetate staining and quantitative real-time polymerase chain reaction, the results revealed that BFR-EVs significantly suppressed ROS generation and oxidative stress induced by H_2_O_2_ and ultraviolet irradiation. Our findings suggest that BFR-EVs hold the potential as a natural candidate for healing chronic skin wounds.

## 1. Introduction

The skin serves as a physical barrier that protects internal organs from the external environment and regulates body temperature, water balance, and electrolyte homeostasis [1]. However, skin damage caused by injury or disease can result in severe physiological imbalances and bacterial infections, potentially leading to significant disability, even death [2]. Repair of damaged skin involves complex biological processes ranging from coagulation/inflammation to re-epithelialization and remodeling. Coagulation, the first step in wound healing, is achieved through formation of a platelet clot and a fibrin matrix. Inflammatory response, the second step in wound healing, is initiated by the recruitment of neutrophils and macrophages [3]. After elimination of invading bacteria by the inflammatory response, proliferation and migration of cells such as fibroblasts begin, followed by deposition of new extracellular matrix (ECM), collagen, and fibronectin [3]. During a wound healing process, low levels of reactive oxygen species (ROS) can promote physiological functions to enhance the normal wound healing process [4].

ROS are bioactive molecules produced during cellular metabolism in aerobic organisms. They play a crucial role in normal wound healing processes [5]. At moderate levels, ROS can promote platelet activation and facilitate the proliferation and migration of epidermal cells by activating the epidermal growth factor receptor and keratinocyte growth factor receptor [6,7]. The proliferation and migration of skin cells, including fibroblasts, are crucial processes for successful skin wound healing [8]. However, in the chronic skin wound environment, the activity of proteases such as matrix metalloproteinases (MMPs) increases, while the activity of protease inhibitors such as tissue inhibitor of MMPs (TIMPs) decreases, promoting degradation of extracellular matrix (ECM) proteins and growth factors [6,9]. Additionally, excessive ROS, a hallmark of chronic skin wounds, cause indiscriminate damage to cellular components such as DNA, lipids, and proteins, leading to the destruction of ECM proteins [10,11]. Consequently, in chronic skin wound lesions, the hyperactivity of MMPs and excessive ROS create an unfavorable proteolytic environment for wound healing, inhibiting the proliferation and migration of skin cells such as fibroblasts and the subsequent wound healing process. During the inflammatory phase, neutrophils and macrophages recruited to wound lesions can secrete a significant amount of ROS, along with inflammatory cytokines. These secreted ROS can directly attack pathogens, thereby preventing wound infection [5,12]. However, repeated exposure to harmful environments, such as ultraviolet (UV) irradiation during the wound healing process, can increase ROS levels and disrupt the antioxidant defense system [13]. Excessive oxidative stress impairs the normal wound healing process by damaging cell membrane proteins and chromosomal integrity, and can even lead to cell death [14,15,16]. Moreover, the prolongation of inflammatory response, a key feature of chronic skin wounds, can result in a massive accumulation of ROS. This accumulation can inhibit the transition from inflammation to re-epithelialization, making skin wound healing more challenging [15]. As a result, many researchers have attempted to use antioxidants to repair chronic skin wounds [17,18,19]. While an excess of ROS within the cells of a wound lesion is not the sole cause for the failure to progress to the repair phase during wound healing, certain studies have indicated that expedited wound healing can occur when antioxidants are employed in tandem with the provision of sufficient oxygen and nutrients through nanomaterials [20,21,22] or with the use of growth factors, such as basic fibroblast growth factor (bFGF) and vascular endothelial growth factor (VEGF) [23]. This suggests that ROS scavenging alone with antioxidants may be insufficient for the treatment of chronic wounds but may have a synergistic effect when used in conjunction with other therapeutic approaches.

Extracellular vesicles (EVs) contain biological molecules, such as nucleic acids, proteins, and lipids, and are secreted during cell-to-cell communication among various cell types [24]. These vesicles are categorized into exosomes, microvesicles, and apoptotic bodies based on their size (50–1000 nm) and biogenesis mechanism [25]. While EVs derived from mammalian cells, including human cells, have been extensively studied, recent reports have highlighted the biological capacities of plant-derived EVs, such as antioxidant and anti-inflammatory effects, as well as pro-angiogenic effects [26,27,28,29]. Similar to mammalian cell-derived EVs, plant-derived EVs also deliver various bioactive substances and facilitate inter-kingdom communication through a lipid bilayer with an aqueous core [30,31]. In addition, plant-derived EVs offer advantages for mass production due to their lower production cost and shorter production time compared to mammalian cell-derived EVs [32]. Accordingly, researchers are exploring methods to isolate a large amount of plant-derived EVs in a relatively simple and efficient manner [33].

Currently, a wide range of techniques, such as ultracentrifugation, differential centrifugation, ultrafiltration, polymer-based precipitation, size exclusion chromatography, and immunoaffinity, are employed for the isolation of EVs from edible plants [34,35]. Among these methods, the polyethylene glycol (PEG)-based precipitation approach offers the benefit of facilely and inexpensively obtaining a large amount of EVs at once [33]. As one of the biocompatible polymers, PEG is utilized in a wide range of pharmaceuticals [36]. Nevertheless, an excessive PEG that remains unresolved during the isolation process poses one of the limitations of the PEG-based isolation technique. Co-precipitated molecules, such as non-vesicle-associated proteins, may diminish the purity of isolated EVs [37,38]. Therefore, recent studies have suggested additional purification steps, such as ultracentrifugation and filtration, to eliminate excess PEG [38,39].

The balloon flower (*Platycodon grandiflorus*) is a perennial plant that belongs to the *Platycodon* L. of the Campanulaceae family and is widely distributed in Northeast Asia [40]. In particular, the balloon flower root (BFR), called doraji in Korea and jiegeng in China, has been consumed as both a food and traditional medicine for treating respiratory diseases such as cough, sore throat, and tonsillitis [41]. Various studies have shown that BFR contains bioactive compounds, including flavonoids, phenolic acids, platycodins, polyacetylenes, and polysaccharides [42]. Of these compounds, platycodins are the major physiologically active components of BFR and are oleanane-type pentacyclic triterpenoid saponins [43]. Recently, platycodins have been found to have anti-inflammatory, anti-tumor, and antioxidant effects [44,45]. Moreover, protective effects of BFR extract against hepatotoxicity and neurotoxicity in animal models have been reported [46,47,48]. The purpose of this study was to isolate EVs from BFRs, which are known to possess bioactive functions, using a PEG-based precipitation and ultracentrifugation technique. Additionally, we aimed to evaluate the therapeutic potential of the BFR-EVs for chronic wound healing using in vitro experimental models. Our results suggest that BFR-EVs hold potential as a natural candidate for healing chronic cutaneous wounds.

## 2. Materials and Methods

### 2.1. Chemicals and Reagents

Paraformaldehyde, phosphotungstic acid, PKH67 green fluorescent dye, lipopolysaccharide (LPS), crystal violet (CV), and 2′,7′-dichlorodihydrofluorescein diacetate (H2DCFDA) were purchased from Sigma-Aldrich (St. Louis, MO, USA). A BCA assay kit, penicillin and streptomycin, trypsin-EDTA, trypan blue solution, and Hoechst 33342 were obtained from Thermo Fisher Scientific (Waltham, MA, USA), and TOPScript™ RT DryMIX, TOPreal™ qPCR 2× PreMIX were acquired from Enzynomics (Daejeon, Republic of Korea). RiboEx™ LS and WST-8 solution were purchased from GeneAll (Seoul, Republic of Korea) and Biomax Inc. (Seoul, Republic of Korea), respectively.

### 2.2. Isolation of BFR-EVs from BFR

BFR was obtained from a local farm. To remove impurities such as soil and dust, the BFR underwent multiple washes with distilled water. Afterward, the BFR was mixed with phosphate-buffered saline (PBS) at a ratio of 1:3 (*w*/*w*) and homogenized using a blender to extract BFR juice. To remove large debris, the BFR juice was sequentially centrifuged at 1000× *g* for 10 min, 3000× *g* for 30 min, and 10,000× *g* for 60 min [27,49]. The supernatant was filtered through a 0.45 μm filter (Hyundai Micro, Seoul, Republic of Korea), and then mixed with a 16% PEG solution at a 1:1 ratio. The mixture was incubated at 4 °C for 16 h, and the resulting samples were centrifuged at 1500× *g* for 30 min. The pellet was subsequently resuspended in PBS, and any excess PEG was removed by ultracentrifugation at 100,000× *g* for 60 min (Beckman Coulter, Brea, CA, USA). Finally, the isolated BFR-EVs was resuspended in PBS and stored at −80 °C until further use.

### 2.3. Characterization of BFR-EVs

The size distribution and concentration of BFR-EVs were analyzed by nanoparticle tracking analysis (NTA, NanoSight NS300, Malvern Panalytical, Malvern, UK) [50]. Briefly, BFR-EVs were appropriately diluted in PBS and injected into a laser chamber using a 1 mL syringe. The total protein concentration of BFR-EVs was measured quantitatively using a BCA assay according to the manufacturer’s instructions. Zeta potential and polydispersity index (PDI) of BFR-EVs were determined with a Zetasizer Nano ZS (Malvern Panalytical, Malvern, UK). Next, the morphology of the BFR-EVs was observed by transmission electron microscopy (TEM). In this process, BFR-EVs were fixed with 2% PFA, dried on carbon-coated copper grids (Ted Pella Inc., Redding, CA, USA), and subsequently stained with 1% phosphotungstic acid. Finally, the stained BFR-EVs were dried on a copper mesh grid for 15 min and observed with a JEM-2100F electron microscope (JEOL, Tokyo, Japan).

### 2.4. Cell Culture

RAW 264.7 cells were acquired from the Korea Cell Lines Bank (Seoul, Republic of Korea) and cultured in Dulbecco’s modified Eagle’s medium (DMEM, Welgene, Daejeon, Republic of Korea) supplemented with 10% fetal bovine serum (DMEM, Welgene, Daejeon, Republic of Korea), 100 U/mL penicillin, and 100 μg/mL streptomycin. The cells were collected with a cell scraper (SPL, Pocheon, Republic of Korea) and were passaged. Human dermal fibroblasts (HDFs, #C-004-5C, Thermo Fisher Scientific) were maintained in the same medium as RAW 264.7 cells. At 70–80% confluence, the HDFs were detached with trypsin-EDTA and were passaged. HDFs under passage number 10 were used in this study in all experiments. All cells were incubated at 37 °C in a humidified atmosphere with 5% CO_2_.

### 2.5. Cytotoxicity Assessment of BFR-EVs

The cytotoxicity of BFR-EVs was determined by trypan blue exclusion assay. Cells were seeded into 24-well plates (SPL) at a density of 1 × 10^4^ cells/cm^2^ and incubated at 37 °C in a humidified atmosphere with 5% CO_2_ for 24 h. After washing with PBS, cells were incubated in medium supplemented with different concentrations of BFR-EVs up to 50 × 10^9^ particles/mL. After incubation for 24 and 48 h, the detached cells were treated with trypan blue. The cell viability was measured by counting the number of viable and dead cells using a hemocytometer (Marienfeld GmbH, Marienfeld, Germany).

### 2.6. Cellular Uptake of BFR-EVs

To confirm cellular uptake of BFR-EVs, BFR-EVs were labeled with PKH67 green fluorescent dye according to the manufacturer’s instructions [29]. HDFs were then incubated in culture medium containing 10 × 10^9^ particles/mL of labeled BFR-EVs. After incubation at 37 °C in a humidified atmosphere with 5% CO_2_ for 12 h, the nuclei of HDFs were stained with 2.5 μg/mL of Hoechst 33342 for 20 min. The cellular uptake of BFR-EVs was then observed by fluorescence microscopy (Leica, Bensheim, Germany).

### 2.7. Anti-Inflammation Activity Assay

To assess the impact of BFR-EVs on pro-inflammatory cytokine gene expression, RAW 264.7 cells were seeded into 24-well plates at a density of 1.5 × 10^5^ cells/cm^2^ and incubated at 37 °C in a humidified atmosphere with 5% CO_2_ for 24 h. After incubation, RAW 264.7 cells were treated with varying concentrations of BFR-EVs for 24 h. To stimulate an inflammatory response, the cells were treated with 50 ng/mL LPS for an additional 24 h. Subsequently, the levels of pro-inflammatory cytokine gene expression in the LPS-stimulated RAW 264.7 cells were determined by quantitative real-time polymerase chain reaction (qRT-PCR).

### 2.8. Quantitative RT-PCR Analysis

Total RNA was extracted from the cells by utilizing RiboEx™ LS, followed by reverse transcription of extracted total RNA to cDNA using TOPScript™ RT DryMIX with a dT 18 plus primer. The specific primers were then combined with the cDNA and TOPreal™ qPCR 2× PreMIX (SYBR Green with low ROX) for qRT-PCR analysis using a qTOWER^3^ machine (Analytik Jena, Jena, Germany). The sequences of specific primers used in this study are summarized in Table 1. Each mRNA expression level was normalized against the expression level of glyceraldehyde 3-phosphate dehydrogenase (GAPDH) as an endogenous control. The relative mRNA expression level in comparison to the control was determined by the 2^−ΔΔCt^ method.

### 2.9. Cell Proliferation Assay

To evaluate impact of BFR-EVs on proliferation of HDFs, water-soluble tetrazolium salt-8 (WST-8) assay was performed. Briefly, HDFs were seeded into 96-well plates (SPL) at a density of 1 × 10^4^ cells/cm^2^ and incubated in culture medium for 24 h. For serum starvation, HDFs were further incubated in DMEM containing 0.5% FBS for 24 h and maintained in serum-free DMEM/F12 medium supplemented with various concentrations of BFR-EVs for 24 h and 48 h. Subsequently, WST-8 solution was added to each well, and the absorbance was measured at 450 nm using a microplate reader after an additional 2 h of incubation. 

### 2.10. Cell Migration Assay

Impact of BFR-EVs on migration of HDFs was assessed using both scratch closure and transwell migration assay. For the scratch closure assay, HDFs were seeded into 24-well plates at a density of 4 × 10^4^ cells/cm^2^ and maintained for 24 h. After a 24 h serum starvation, a sterile pipette tip was used to scratch the cell monolayer. Cells were washed several times with PBS to remove cell debris and then incubated in DMEM/F12 containing various concentrations of BFR-EVs for a specific time period. The scratched area was subsequently observed under an optical microscope at each time point. Transwell migration assay was performed as previously described [26]. Briefly, HDFs were seeded onto membranes in the upper chamber of a transwell plate (Corning, Glendale, AZ, USA) with 8.0 μm pores at a density of 4 × 10^4^ cells/cm^2^ and incubated for 24 h. The cells were then incubated in serum-free DMEM/F12 supplemented with BFR-EVs at each concentration for 24 h to induce migration to the bottom surface of the membrane. After fixation with 4% PFA, the cells remaining on the top of the membrane were removed with a cotton swab, and the cells that migrated to the bottom of the membrane were stained with 0.5% CV for 15 min. The CV-stained membrane was immersed in 50% acetic acid to dissolve the CV, and the optical absorbance at 560 nm was measured using a microplate reader for quantitative analysis of CV staining.

### 2.11. Antioxidant Activity Assay

The antioxidant effect of BFR-EVs was evaluated by H2DCFDA staining and qRT-PCR analysis. Briefly, HDFs were seeded into 6-well plates (SPL) at a density of 1.5 × 10^4^ cells/cm^2^ and cultured at 37 °C in a humidified atmosphere with 5% CO_2_ for 24 h. HDFs were then treated with different concentrations of BFR-EVs in serum-free DMEM/F12 for 24 h. Intracellular ROS were induced by H_2_O_2_ treatment and UVB (315 nm) irradiation. To generate H_2_O_2_ induced ROS, cells were washed with PBS several times and treated with 0.5 mM H_2_O_2_ for 3 h. After 3 h of H_2_O_2_ treatment, a specific assay was performed. For UVB-induced ROS generation, cells were washed with PBS several times and 700 μL of PBS was added to each well. Cells were then irradiated with 80 mJ/cm^2^ of UVB generated by a Bio-Sun illuminator (Vilber Lourmat, Eberhardzell, Germany). After UV irradiation, cells were maintained in serum-free DMEM/F12 for 24 h before a specific assay. Specific analysis was then performed after washing the cells several times with PBS. Intracellular ROS levels were observed by H2DCFDA staining. Cells were treated with 10 μM H2DCFDA for 30 min. Cell nuclei were stained with 2.5 μg/mL Hoechst 33342 for 20 min. Subsequently, the cells were washed three times with PBS. Intracellular ROS levels were analyzed using a fluorescence microscope and a flow cytometer (FACSymphony™ A3, BD, San Jose, CA, USA).

### 2.12. Statistical Analysis

All results presented in this article are expressed as the mean value accompanied by the corresponding standard deviation. Statistical significance between the experimental groups was determined through one-way analysis of variance (ANOVA), followed by Tukey’s post hoc test. The criterion for statistical significance was based on a *p*-value of less than 0.05. All quantitative data were obtained from a minimum of three independent samples, representing the experiments conducted several times.

## 3. Results and Discussion

### 3.1. Isolation and Characterization of BFR-EVs

**Figure 1:**
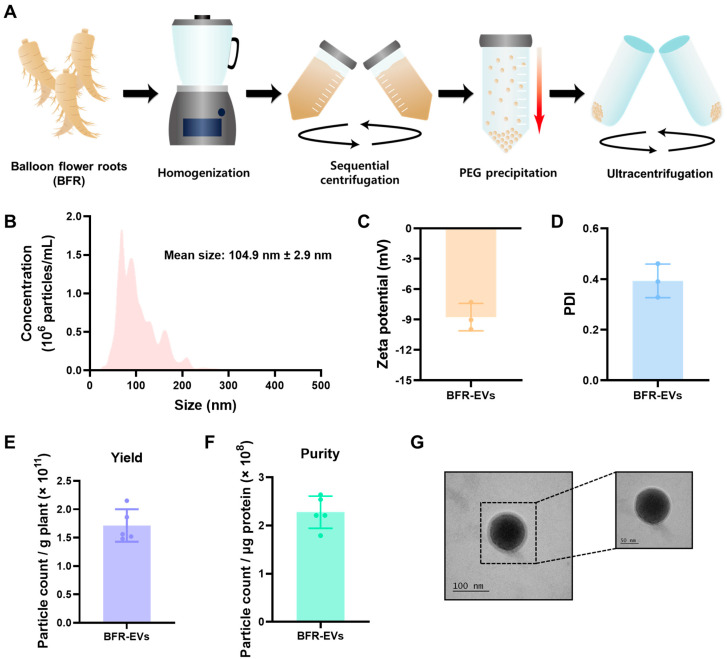
Isolation and characterization of balloon flower root-derived extracellular vesicles (BFR-EVs). (**A**) Schematic illustration of BFR-EVs isolation procedure. (**B**) Size distribution and average size of BFR-EVs measured by nanoparticle tracking analysis (NTA). BFR-EVs showed a major peak at 60 to 120 nm with an average size of 104.9 nm. (**C**,**D**) Zeta potential and polydispersity index (PDI) value of BFR-EVs determined by dynamic light scattering (DLS). (**E**) Production yield of BFR-EVs calculated by dividing the total number of isolated BFR-EVs by BFR mass. (**F**) Purity of BFR-EVs represented as the count of particles-to-microgram of total protein. (**G**) Transmission electron microscopy image of isolated BFR-EVs.

PEG-based precipitation is a technique commonly used for isolating EVs from various cell cultures due to its ability to handle large-scale samples at low cost [51]. It has also been utilized for the isolation of plant-derived EVs, which requires processing a significant quantity of samples simultaneously. Nevertheless, excessive PEG that remains during the isolation process may restrict the practical application of the isolated EVs. We efficiently isolated EVs from BFR using a PEG-based precipitation method, followed by ultracentrifugation to eliminate any remaining excess PEG (Figure 1A). In our previous study, we investigated the optimal PEG-based precipitation method for isolating EVs from *Aloe saponaria* peels and found that reducing the PEG concentration and precipitation time improves the purity of EVs [26]. According to the NTA analysis of BFR-EVs isolated through precipitation with 8% PEG and ultracentrifugation, the BFR-EVs exhibited a major peak at 60 to 120 nm with an average size of 104.9 nm (Figure 1B). Zeta potentials and PDI of the BFR-EVs were further evaluated by dynamic light scattering (DLS) analysis. The results revealed that the average zeta potential of the BFR-EVs was −8.76 mV, and their PDI value was 0.39 (Figure 1C,D). Next, we evaluated the production yield and purity of BFR-EVs based on the number of BFR-EVs measured by NTA. The production yield of BFR-EVs, which was determined by dividing the number of isolated BFR-EVs by the weight of BFR used, was 1.71 × 10^11^ particles per one gram of BFR (Figure 1E). The purity of BFR-EVs, calculated by dividing the number of BFR-EVs by micrograms of total protein measured by BCA assay, was 2.27 × 10^8^ particles/mL (Figure 1F). Finally, TEM images confirmed that these BFR-EVs had a spherical morphology with a size of approximately 100 nm, consistent with NTA results (Figure 1G).

### 3.2. Cytotoxic Effect and Cellular Uptake of BFR-EVs

**Figure 2:**
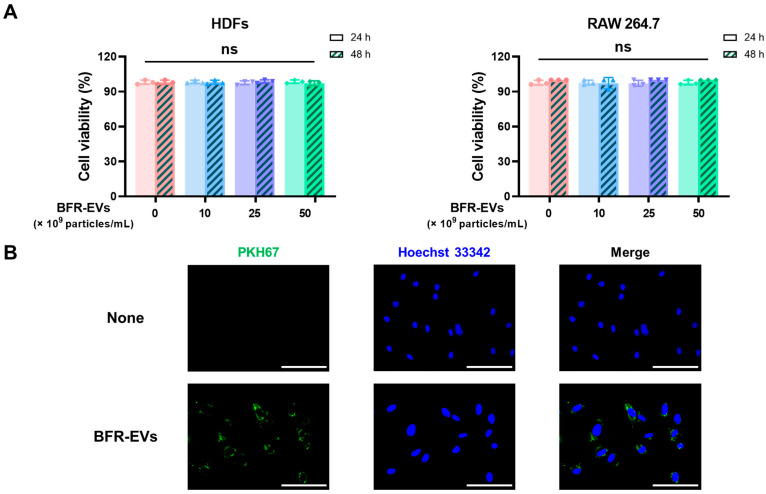
Cytotoxicity and cellular uptake of BFR-EVs. (**A**) Cell viability of BFR-EVs in human dermal fibroblasts (HDFs) and RAW 264.7 cells measured by trypan blue exclusion assay. No significant cytotoxicity was observed, even when cells were treated with BFR-EVs above therapeutic doses (ns: not significant, *n* = 3). (**B**) Uptake of BFR-EVs into HDFs. PKH67 green fluorescent dye-labeled BFR-EVs were added to the culture medium and incubated for 12 h. The HDFs were then observed under a fluorescence microscope (scale bar = 100 μm).

In order to assess the cytotoxicity of BFR-EVs, we treated HDFs and RAW 264.7 cells with BFR-EVs at different concentrations up to 50 × 10^9^ particles/mL for 24 and 48 h. Subsequently, cell viability was measured by trypan blue exclusion assay. Given the low cytotoxicity of EVs derived from various edible plants [27,49], we postulated that BFR-EVs would also demonstrate bio-compatibility. As expected, no significant cytotoxicity was observed, even at doses higher than the therapeutic dose used in this study (Figure 2A). Next, to validate cellular uptake of BFR-EVs, we labeled them with the green fluorescent dye PKH67 and treated HDFs with 10 × 10^9^ particles/mL of labeled BFR-EVs for 12 h. Under fluorescence microscopy, HDFs displayed green fluorescence, indicating the cellular uptake of BFR-EVs (Figure 2B). These results collectively suggest that BFR can efficiently deliver bioactive components derived from balloon flower root, such as phytochemicals and proteins, to cells without eliciting significant cytotoxicity.

### 3.3. Suppression Effect of BFR-EVs on Expression of Pro-Inflammatory Cytokines

**Figure 3:**
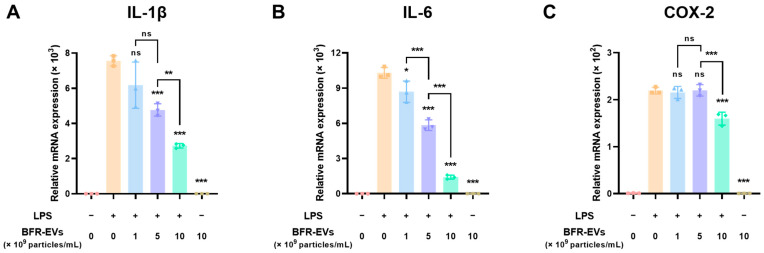
Suppression of pro-inflammatory cytokine gene expression by BFR-EVs in lipopolysaccharide (LPS)-stimulated RAW 264.7 cells. (**A**–**C**) The mRNA expression levels of pro-inflammatory cytokines in BFR-EVs-treated cells were quantitatively analyzed by qRT-PCR. BFR-EVs significantly reduced the expression of IL-1β (**A**), IL-6 (**B**), and COX-2 (**C**) induced by LPS stimulation as compared to the control group not treated with BFR-EVs (* *p* < 0.05, ** *p* < 0.01, *** *p* < 0.005, ns: not significant, *n* = 3).

In the process of normal wound healing, inflammation plays a vital role in promoting the proliferation and migration of cells by eliminating foreign substances that have infiltrated the body [52]. However, prolonged inflammation can elevate the ROS levels in the wound lesion, which can interfere with normal wound healing and result in chronic skin wounds [14,15]. It should be noted that excessive ROS in wound lesions not only cause oxidative stress, but also activate pro-inflammatory genes, thereby aggravating chronic skin wounds [53,54,55]. Several studies have reported that the use of naturally derived or chemically synthesized antioxidants inhibits inhibitory responses and stimulates the repair of chronic wounds, including diabetic wounds [56,57]. To explore the potential of BFR-EVs for chronic wound healing, we first evaluated their ability to suppress pro-inflammatory cytokine gene expression. To this end, RAW 264.7 cells, an immortalized mouse macrophage cell line, were treated with BFR-EVs at a concentration up to 10 × 10^9^ particles/mL. An inflammatory response was then induced by LPS treatment. Next, mRNA expression levels of pro-inflammatory cytokines, such as interleukin (IL)-1β, IL-6, and cyclooxygenase-2 (COX-2), in non-treated RAW 264.7 cells and those treated with BFR-EVs were determined using qRT-PCR analysis. As shown in Figure 3A,B, LPS treatment led to an over 6000-fold and 9000-fold increase in the mRNA expression levels of IL-1β and IL-6, respectively. However, treatment with BFR-EVs reduced mRNA expression levels of IL-1β and IL-6 by more than 60%, with a significant decrease in IL-6 mRNA levels in a dose-dependent manner. Similarly, the mRNA expression level of COX-2, which mediates the inflammatory response and oxidative stress induced by LPS, was reduced by more than 25% after treatment with 10 × 10^9^ particles/mL of BFR-EVs (Figure 3C). These results suggest that BFR-EVs can induce a transition from excessive and sustained inflammatory responses to re-epithelialization by suppressing the expression of pro-inflammatory cytokines.

### 3.4. Promotion Effects of BFR-EVs on Proliferation and Migration of HDFs

**Figure 4:**
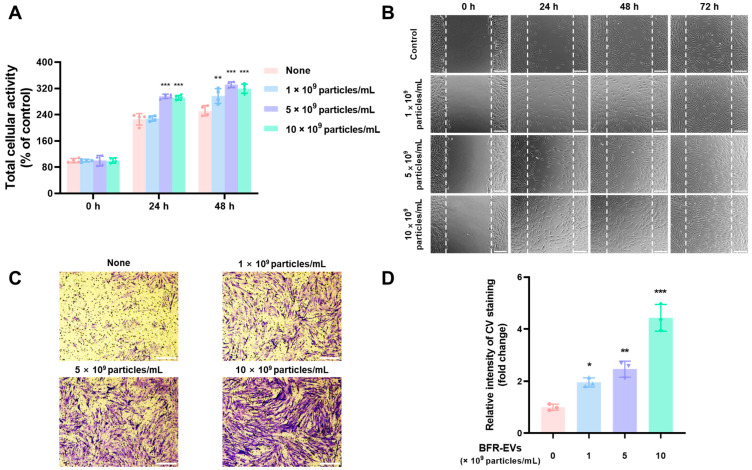
Promotion of proliferation and migration by BFR-EVs in HDFs. (**A**) HDFs were serum-starved and then treated with BFR-EVs for 24 and 48 h in serum-free medium, after which the population of viable HDFs was measured by WST-8 assay. Statistical significance was determined by comparison with the control group not treated with BFR-EVs at each time point, unless otherwise noted (** *p* < 0.01, *** *p* < 0.005, *n* = 4). (**B**) Scratch closure assay with HDFs treated with BFR-EVs (scale bar = 200 µm). The dashed lines indicate the initial scratched area. (**C**) Transwell migration assay with HDFs treated with BFR-EVs (scale bar = 200 µm). After treatment with BFR-EVs for 24 h, HDFs that migrated to the bottom side of the porous membrane were stained with CV and observed under an optical microscope. (**D**) Quantitative analysis of a transwell migration assay, with crystal violet (CV)-stained membrane cut out and immersed in a 50% acetic acid solution to dissolve crystalized CV. The amount of stained CV was measured as optical absorbance at 560 nm. Statistical significance was represented by comparison with the non-treated control group (* *p* < 0.05, ** *p* < 0.01, *** *p* < 0.005, *n* = 3).

Next, we evaluated the effects of BFR-EVs on the proliferation and migration of HDFs located in dermal tissues. After serum starvation to minimize the stimulatory effect induced by serum, HDFs were treated with different concentrations of BFR-EVs ranging from 1 to 10 × 10^9^ particles/mL. Subsequently, total cellular activity of HDFs was measured using a WST-8 assay. High doses (5 and 10 × 10^9^ particles/mL) of BFR-EVs promoted HDF proliferation after 24 h of treatment, while a low dose (1 × 10^9^ particles/mL) promoted cell proliferation after 48 h of treatment (Figure 4A). Next, we conducted a scratch assay to assess the effect of BFR-EVs on HDF migration. HDFs were scratched and treated with varying concentrations of BFR-EVs after serum starvation. As shown in Figure 4B, HDFs treated with BFR-EVs closed the gap faster than untreated HDFs. For HDFs treated with BFR-EVs at a concentration of 10 × 10^9^ particles/mL, the gap had already been filled after 48 h. We further evaluated the effect of BFR-EVs on HDF migration through a transwell migration assay. To end this, HDFs were seeded onto the upper side of the porous membrane and further incubated with BFR-EVs. After 24 h of incubation, HDFs that had migrated to the opposite side were stained with CV and observed using an optical microscope. The images of CV staining revealed that HDF migration was significantly promoted by treatment with BFR-EVs in a dose-dependent manner (Figure 4C). Quantitative analysis of CV staining also demonstrated the stimulatory effect of BFR-EVs on HDFs migration, with HDFs treated with 10 × 10^9^ particles/mL of BFR-EVs showing more than a 4-fold increase in migration compared to non-treated HDFs (Figure 4D). Our findings suggest that BFR-EVs have the potential to enhance the proliferation and migration of HDFs, which can be harnessed for treating chronic wounds.

### 3.5. Antioxidant Effect of BFR-EVs on Oxidative Stress Induced by UV Irradiation

**Figure 5:**
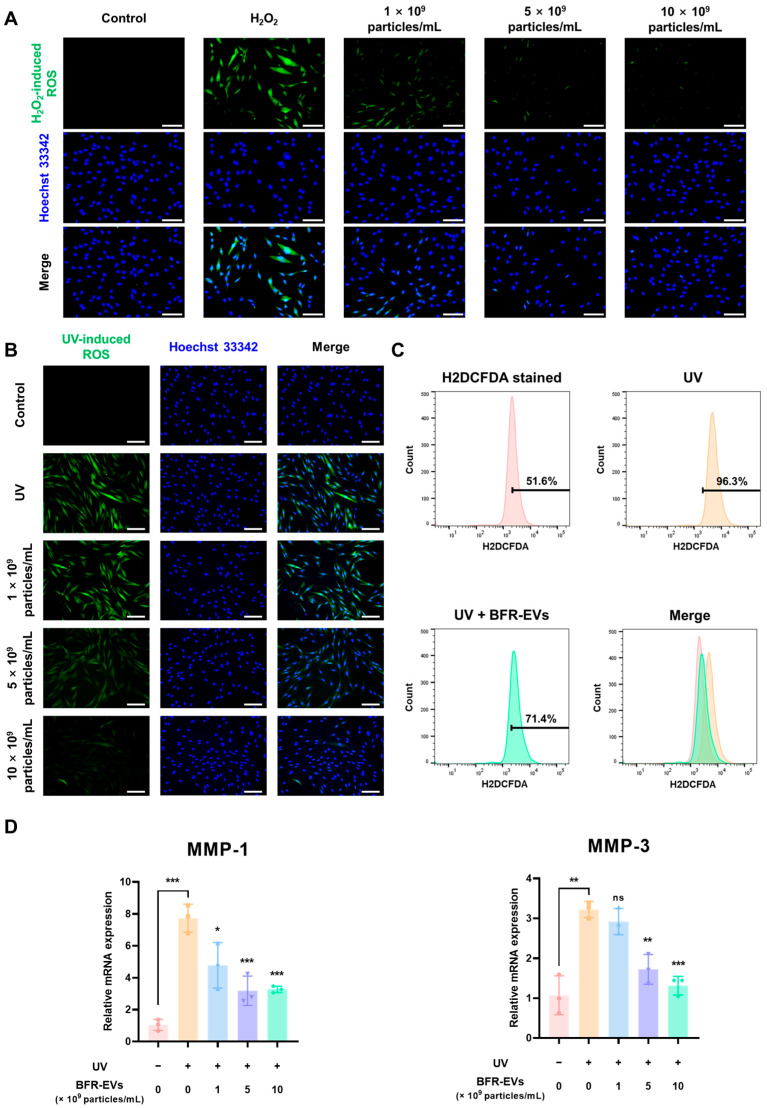
Inhibition of reactive oxygen species (ROS) generation by BFR-EVs in H_2_O_2_ or ultraviolet B (UVB)-treated HDFs. (**A**) H_2_O_2_-induced intracellular ROS in HDFs treated with BFR-EVs observed through H2DCFDA staining. After 24 h of treatment with BFR-EVs, intracellular ROS levels induced by 0.5 mM H_2_O_2_ and nuclei of HDFs were stained with H2DCFDA and Hoechst 33342 and observed under a fluorescence microscope (scale bar = 200 µm). (**B**) UVB-induced intracellular ROS in HDFs treated with BFR-EVs observed through H2DCFDA staining. After 24 h of treatment with BFR-EVs, intracellular ROS levels induced by 80 mJ/cm^2^ of UVB and nuclei of HDFs were stained with H2DCFDA and Hoechst 33342 and observed under a fluorescence microscope (scale bar = 200 µm). (**C**) Flow cytometric analysis of UVB-induced intracellular ROS stained with H2DCFDA. (**D**) Oxidative stress-related mRNA expression levels in UVB-irradiated HDFs were quantitatively analyzed by qRT-PCR. BFR-EVs decreased the expression of UVB-induced MMP-1 and MMP-3. Statistical significance was determined by comparison with the control group not treated with BFR-EVs (* *p* < 0.05, ** *p* < 0.01, *** *p* < 0.005, ns: not significant, *n* = 3).

Exposure to harmful environments repeatedly or prolonged inflammation due to abnormal inflammatory responses can result in excessive ROS generation in wound lesions, ultimately leading to chronic skin wound. Thus, we investigated the scavenging effect of BFR-EVs on excessive intracellular ROS. To induce ROS generation, we treated HDFs with 0.5 mM H_2_O_2_ for 3 h and evaluated the intracellular ROS levels using H2DCFDA staining. As shown in Figure 5A, HDFs treated with H_2_O_2_ showed elevated levels of ROS, which were dose-dependently scavenged by BFR-EVs. These finding suggest that BFR-EVs possess an antioxidant effect and the ability to protect HDFs from H_2_O_2_-induced oxidative stress through ROS scavenging.

The sunlight that reaches the Earth’s surface comprises UV radiation, which is a hazardous environmental factor that can be easily encountered. There are three types of UV, namely, UVA (320–400 nm), UVB (280–320 nm), and UVC (200–280 nm). The ozone layer obstructs UVC, whereas UVA and UVB can reach the skin and induce the generation of ROS [58]. UVB is believed to be the most harmful UV ray since it directly and indirectly generates ROS in irradiated cells and causes DNA mutation by producing cyclobutane pyrimidine dimers and other photoproducts [59]. To evaluate the antioxidant properties of BFR-EVs, which could protect HDFs from UVB-induced oxidative stress, we treated HDFs with different concentrations of BFR-EVs for 24 h. Then, the intracellular ROS induced by 80 mJ/cm^2^ of UVB in HDFs was visualized using H2DCFDA staining. HDFs exposed to UVB exhibited substantial intracellular ROS generation, which was also scavenged by BFR-EVs in a dose-dependent manner, similar to H_2_O_2_-induced ROS (Figure 5B). Flow cytometry also revealed the inhibitory effect of BFR-EVs on intracellular ROS generation, where the treatment with 10 × 10^9^ particles/mL of BFR-EVs induced a shift from a population with UV-induced high ROS levels to a population with low ROS levels (Figure 5C). Additionally, we investigated the impact of BFR-EVs on oxidative stress-related gene expression in UVB-irradiated HDFs. MMPs are enzymes that can cleave almost all ECM proteins as well as several non-ECM proteins [60]. Excessive ROS in wound lesions induced by harmful environmental factors such as UV irradiation can mediate overactivation of MMPs, resulting in interference with normal wound healing [61,62]. Among various MMP enzymes, MMP-1 is the most commonly expressed subtype, primarily cleaving collagen, a major structural component of the dermis. MMP-3 not only activates latent MMP-1, but also degrades structural glycoproteins and proteoglycans [61,62]. UVB irradiation elevated mRNA expression levels of MMP-1 and MMP-3 in HDFs by more than 7-fold and 3-fold, respectively. However, treatment with 10 × 10^9^ particles/mL of BFR-EVs significantly reduced mRNA expression levels of both MMP-1 and MMP-3 enzymes (Figure 5D). Taken together, our results suggest that BFR-EVs can suppress ROS generation caused by UV irradiation, which is an easily encountered harmful environmental factor, and modulate oxidative stress-related gene expression.

## 4. Conclusions

Recently, several studies have reported on the physiological activities of EVs isolated from various plants [13,29,49]. These plant-derived EVs contain phytochemicals, nucleic acids, and proteins, which are physiologically active molecules found in plants. In this study, we isolated EVs from BFR and evaluated their potential for the treatment of chronic wounds. The PEG-based precipitation method enables the isolation of a large quantity of EVs in a rapid and cost-effective manner, while ultracentrifugation aids in the removal of excess PEG, making the isolated EVs more suitable for further applications. BFR-EVs isolated using these two methods were effectively delivered intracellularly, with no observed cytotoxicity. Furthermore, intracellularly delivered BFR-EVs downregulated the expression of pro-inflammatory cytokine genes and promoted the proliferation and migration of HDFs. Notably, BFR-EVs significantly inhibited H_2_O_2_ and UVB-induced ROS generation and oxidative stress in HDFs. These findings are particularly noteworthy, as various compounds that display antioxidant activity in vitro have demonstrated efficacy in treating cutaneous wounds when applied in vivo. Therefore, our results suggest that BFR-EVs, obtained using a combination of these two EVs isolation methods, hold promise as natural candidates for treating chronic skin wounds.

## Figures and Tables

**Table 1:** List of specific primers used for qRT-PCR.

Gene (Accession #)	Primer	Sequence (5′-3′)
Mouse GAPDH (NC_000072.7)	Sense	GTC AGT GGT GGA CCT GAC CT
	Antisense	TGC TGT AGC CAA ATT CGT TG
IL-6 (NC_000007.14)	Sense	GCT ACC AAA CTG GAT ATA ATC GGA
	Antisense	CCA GGT AGC TAT GGT ACT CCA GAA
IL-1β (NC_000002.12)	Sense	AGT TGA CGG ACC CCA AAA G
	Antisense	AGC TGG ATG CTC TCA TCA GG
COX-2 (NC_012920.1)	Sense	GGG CTC AGC CAG GCA GCA AAT
	Antisense	GCA CTG TGT TTG GGG TGG GCT
Human GAPDH (NC_000012.12)	Sense	GTC AGT GGT GGA CCT GAC CT
	Antisense	TGC TGT AGC CAA ATTCGT TG
MMP-1 (NC_000011.10)	Sense	CAT CGT GTT GCA GCTCAT GA
	Antisense	ATG GGCTGG ACA GGATTT TG
MMP-3 (NC_000011.10)	Sense	TGC TGC TCA TGAAAT TGG CC
	Antisense	TCA TCT TGA GACAGG CGG AA

## Data Availability

The original contributions presented in the study are included in the article.
